# Promising behavior change techniques for climate-friendly behavior change – a systematic review

**DOI:** 10.3389/fpubh.2024.1396958

**Published:** 2024-08-12

**Authors:** Lisa Masciangelo, Susanne Lopez Lumbi, Michel Rinderhagen, Claudia Hornberg, Michaela Liebig-Gonglach, Timothy Mc Call

**Affiliations:** ^1^Department of Sustainable Environmental Health Sciences, Medical School OWL, Bielefeld University, Bielefeld, Germany; ^2^Department of Environment and Health, Bielefeld School of Public Health, Bielefeld University, Bielefeld, Germany

**Keywords:** sustainability, mitigation, intervention, household, energy use, water use, mobility, BCT

## Abstract

**Introduction:**

Besides societal and governmental actions to mitigate greenhouse gases, individual behavioral changes are also urgently needed to limit global temperature rise. However, these individual changes have proven to be difficult to achieve in the general population.

**Methods:**

We conducted a systematic review in five electronic databases with the aim of systematically depicting the content of interventions that promote climate-friendly behavior in individuals and households in high- and upper-middle-income countries.

**Results:**

We included 25 studies. The analyses included identification of the used Behavior Change Techniques (BCTs) and comparison of their promise ratio. Across our three outcome categories energy consumption, water consumption, and mobility the most frequently used BCT categories are not the ones that are most promising in terms of behavior change.

**Discussion:**

Based on these results, our recommendation for climate change mitigation interventions is to include components that provide concrete instructions on how to perform the desired behavior *(shaping knowledge)*, setting goals and commitments *(goals and planning)*, substituting undesired behavior, and practicing desired behavior *(repetition and substitution)*. Other reviews with similar aims use different wordings, definitions, or degrees of detail in their intervention component labelling which makes it difficult to compare the results. We recommend to use a standardized classification system, like the BCT taxonomy in combination with the promise ratio, which this study has shown to be a suitable tool to classify applied intervention techniques and give an indication of successful techniques.

## 1 Introduction

The anthropogenic climate change is a global crisis with serious implications for public health and therefore requires appropriate action strategies, otherwise the impact on public health and the health of the planet will escalate in the near future (1). Climate change implications, like long periods of heat and drought, or heightened regional frequency and magnitude of precipitation and storms will have a lasting impact on land use, food security, and food production systems (1–4). The associated multiple health risks to the world’s population are already measurable today, in both low- and high-income countries (5).

Climate change mitigation and adaptation are the most important strategies to address current and future climate change implications. The primary goal of mitigation interventions is to reduce anthropogenic emissions or enhance the decrease of greenhouse gases, either on a societal or governmental level (1). Those actions to reduce carbon-emissions are a particularly effective approach to curbing greenhouse gases. However, far-reaching individual behavioral changes are urgently needed to achieve the goal of limiting global temperature rise as well. Lifestyle changes of individuals or communities can be implemented to curb carbon emissions, such as reducing daily car use, switching to green energy sources, or reducing energy use, meat and overall consumption. There are indications that households are responsible for about 72% of the global greenhouse gas emissions (6). The ability to take effective action to mitigate climate change is unevenly distributed among populations (7). In particular households of high-income countries like the United States and most Western European countries are major contributors to the emission of greenhouse gases and therefore an important target group for interventions (6, 8). Analyses show that more than 60% of global greenhouse gas emissions stem from high income countries in North America and Western Europe (9, 10). It is suggested that practice, policy and research should focus on behaviors associated with the greatest greenhouse gas emissions, i.e., mobility, housing and food (10).

Despite the knowledge in the general population about the causes and risks of climate change, sustainable behavioral changes seem difficult to achieve due to various barriers like strong habits and convenience (11). To date, a large number of intervention studies have been published that address different target groups and deal with changing of various aspects of climate-relevant behavior. Not only do the intervention concepts differ considerably in terms of their objectives and strategies but they also vary greatly in terms of their effectiveness (12). Particularly promising and reproducible intervention approaches that lead to a sustainable, climate-friendly behavioral change have not yet been identified. The majority of published interventions use a variety of stimuli which leads to difficulties in identifying the main factor for effectiveness (13). The content and techniques of successful interventions hence needs to be examined more closely and systematically (13). Therefore, one aim was to identify effective household interventions for climate-friendly behavior and their components (i.e., applied behavior change techniques).

The conceptualization of human behavior has been part of research over decades. There are many models that present influencing factors and therefore how to possibly change behavior, i.e., Theory of Planned Behavior, Social Cognitive Theory, Transtheoretical Model among others. The influencing factors presented and validated in those models can help to determine the various social and psychological components that affect behavior and accordingly develop or identify effective intervention strategies. Behavioral theories are seen as the crucial starting points of every intervention. Strategies developed to tackle behavioral patterns are addressing the so-called mechanism of action which are constructs from behavioral theories (14). One approach to systematize intervention strategies is the Behavior Change Technique Taxonomy (BCTTv1) developed by Michie et al. (15, 16). This Behavior Change Techniques (BCTs) classification system was established to ensure a universally valid nomenclature and standardized language in the description of intervention methods that allows to specify, implement, evaluate and replicate complex behavior change interventions. Complex interventions are defined by one or more of the following characteristics: a high number of and interactions between intervention components, a high number and difficulty of behaviors required by those delivering or receiving the intervention, a high number of groups or organizational levels targeted by the intervention, and a high number and variability of outcomes (17). The theoretical framework of the BCTTv1 is the Behavior Change Wheel by Michie et al. (18) which is based on the assumption that a person’s behavior is based on the factors capability, opportunity, and motivation. One or more of them has to change in order to change a behavior. Michie et al. (18) developed nine intervention functions for this to choose from depending on the nature of the targeted behavior. These functions can be translated into the specific BCT for changing behavior (19). The BCTTv1 has already been applied in many review studies that have focused primarily on interventions to promote health-related behavioral changes, e.g., to reduce sedentary behavior or promote physical activity in the context of various diseases (20–22). But, to our knowledge none of the previous systematic reviews with a focus on intervention strategies to promote climate-friendly behaviors (12, 23–26) has systematically classified the components of interventions based on the BCTTv1. Therefore, the second aim of this review was to analyze whether certain BCTs or combinations of different BCTs are more frequently used. To get an indication of the relative effectiveness of each BCT, a promise ratio was calculated as a quotient of a BCT’s frequency in promising and non-promising interventions (20).

The aim of any intervention that promotes climate-friendly behavior is to change current behaviors, and it is thus important that the BCTs that lead to behavioral changes are identified. A systematic recording of techniques and concepts can help to better understand how interventions can lead to climate-friendly behavior. Moreover, it may ensure that successful measures are reproducible. In addition, needs-based measures can be developed for specific population groups in order to achieve a sustainable impact.

## 2 Materials and methods

### 2.1 Identifying and selecting studies

We searched five databases in the fields of Medicine, Psychology, Geography, Public Health, and Ecology, namely MEDLINE (PubMed), PsycINFO, CINAHL, Embase, and Web of Science. Searches were limited to the languages English and German and the time period of the last 15 years (2007 to 2022). The search was conducted between 10/20/2022 and 10/21/2022 by LM using the search terms presented in Table 1. The reporting follows the PRISMA statement for reporting systematic reviews and meta-analyses (28).

**Table 1:** Search terms used in the electronic search using PICO (27, 28).

Population	Intervention (component I)	Intervention (component II)	Outcome
Word group I	Word group II	Word group III	Word group IV
Individual	Climate change^1,2,3,4^	Intervention^2^	Mitigation^4^
Individuals	Climate crisis	Interventions	Adaptation^2,3,4^
Household^4^	Global warming^1,2^	Programme	Pro-environmental behavio(u)r
Households	Greenhouse gas^4^	Programmes	Pro environmental behavio(u)r^2,4^
	Greenhouse gases^1,3^	Program	Environmental behavio(u)r
	Environment^1,2,3,4^	Programs	Sustainable behavio(u)r
	Environmental		Green behavio(u)r
	Carbon footprint^1,3,4^		Behavio(u)r change^2,4^
			Behavio(u)r changes
			Behavio(u)ral change
			Behavio(u)ral changes^3^
			Behavio(u)r modification^2,3,4^
			Behavio(u)ral modification
			Risk reduction behavio(u)r

Terms within word groups combined using “or”; word groups combined using “and”. Title/abstract search filter used for all terms. Subject Headings: ^1^Medline (PubMed) (MeSH terms). ^2^PsycINFO (APA thesaurus of psychological index terms). ^3^CINAHL (CINAHL Subject Headings). ^4^Embase (Emtree terms).

Table 2 presents the inclusion and exclusion criteria. We included interventions that aim at behavior change in climate change mitigation or adaptation. Eligible studies measure quantifiable effects and focus on households or individual adults over the age of 18 years in upper middle- or high-income countries. Due to comparability, we excluded studies that reported on a category that was not reported in other eligible studies and studies that reported aggregated scores across different categories. Further, due to transferability, recycling behavior was excluded because of its country-specific framework conditions that would make transferability difficult.

**Table 2:** Inclusion and exclusion criteria.

Component	Inclusion criteria	Exclusion criteria
Intervention	Interventions that aim at climate change mitigation and adaptation behavior change	Industry interventions; policy interventions; municipality interventions; agricultural interventions; plants, animals
Outcome	Quantifiable effects (behavior change; CO_2_ emission reduction)	All non-quantifiable effects (such as Interviews, missing pre−/ post comparison or no control−/ reference-group) Aggregated scores across different outcome categories (due to comparability) Studies reporting on a category (e. g. flood adaptation or clothing sufficiency) no other eligible studies report on (due to comparability) Studies reporting on recycling behavior (due to transferability)
Target group	Individuals over 18 or households	Children below 18; industry; policy institutions; municipalities; college/university students (because they often live in dormitories where they do not have the same opportunities to implement climate mitigation or adaptation measures as other households)
Setting	High-income countries, upper middle-income countries (29)	Lower middle-income countries, low-income countries (29); workplaces
Study design	Primary studies with all types of study designs reporting quantifiable effects (e.g., intervention studies with and without comparison groups, including RCTs, non-randomised controlled studies, single group studies with post, pre-post, or interrupted time series measurements, case studies)	Secondary studies; qualitative studies

**Figure 1:**
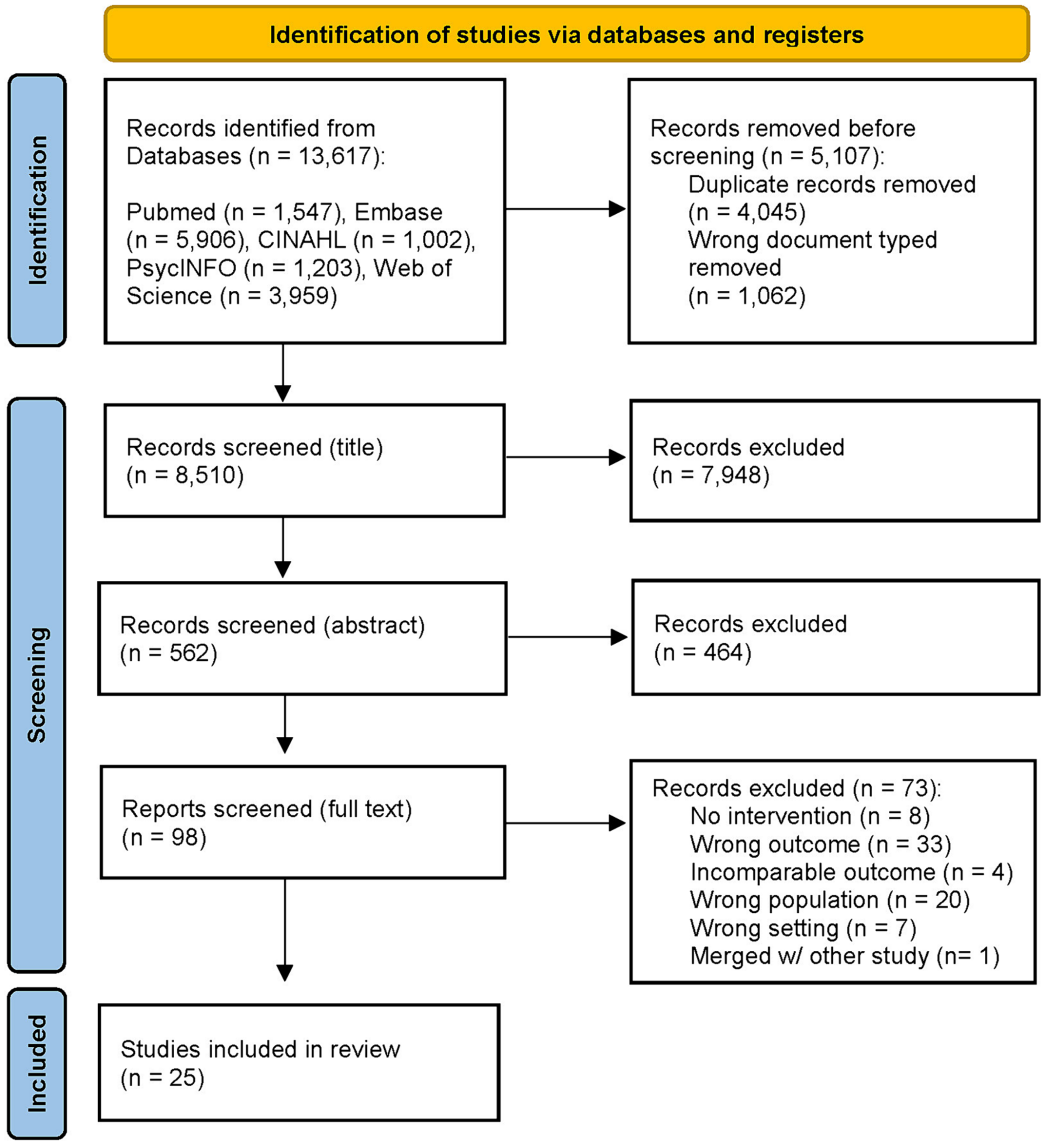
PRISMA flow diagram of study selection process [layout following Page et al. (28)].

The screening process (Figure 1) was conducted in teams of two authors. It started with title screening and disagreement led to inclusion of the study for the following abstract and full-text screening.

### 2.2 Data extraction

From the included studies we extracted information regarding study characteristics (authors, year of publication, location of study (country and continent), sample size, mean age, sex/gender, and socio-economic status), as well as information regarding intervention characteristics (BCTs, BCT categories, outcome category, primary outcomes, results for primary outcomes, and theory basis) [all authors]. For each study two authors independently extracted information from the papers into an Excel sheet (LM, SLL, MR, MLG, TMC). The information was later merged by one of the authors.

### 2.3 Quality assessment

The methodological quality of the included studies was assessed using the Effective Public Health Practice Projects (EPHPP) quality assessment tool for quantitative studies (30). Based on the framework, two authors, TMC and MR, independently assessed each of the studies in the domains 1: selection bias, 2: study design, 3: confounders, 4: blinding, 5: data collection methods, and 6: withdrawals and drop-outs. Discrepancies were discussed and resolved. The overall study quality ratings were determined based on the individual domain ratings in consultation with the author SLL. Depending on the number of domains that were rated as “weak”, the overall study rating was either “weak” (two or more “weak” domain ratings), “moderate” (one “weak” domain rating), or “strong” (no “weak” domain ratings). The description and assessment scheme of the individual domains and the overall rating are publicly available on the EPHPP website.

### 2.4 Behavior change technique taxonomy and promise ratio

To identify and compare the used techniques to achieve behavior change in the interventions we used the Behavior Change Technique Taxonomy v1 (BCTTv1). It provides standardized definitions for intervention components aimed at changing study participants’ behavior (15, 31). Coding was conducted independently by two authors, LM and SLL (32). All text sections describing the interventions in the included studies were scanned. The relevant text sections were then imported into a spreadsheet and assigned to BCT. After coding the first five publications, rules, definitions, and ambiguities were discussed and clarified. Finally, the codes were compared and final agreements were made.

The frequency of the used BCTs in each included intervention was counted [LM]. The relationship between the number of BCTs used and the promise rating of interventions was analyzed with a one-way ANOVA and the (very and quite) promising interventions were contrasted with the non-promising interventions by means of planned comparisons [MR]. As Levene’s test indicated homogeneity of variances, no adjustments were made to the results. A promise ratio was calculated to give an idea of the respective contribution of a specific BCT to the effectiveness of the intervention [SLL, MLG]. The promise ratio is an indicator that has been used before for this purpose (21, 22, 33) and results from the quotient of the frequency of a BCT in the (very and quite) promising interventions and the frequency in non-promising interventions (20). According to its developers Gardner et al. (20) we classified interventions as “very promising” if there was a significant effect on at least one indicator in the pre-post comparison of the intervention group. This effect also had to be greater than in a comparison group. Interventions were classified as “quite promising” if either a significant pre-post effect or a significant effect compared to a comparison group was found. Interventions were classified as “non-promising” if there was neither a significant pre-post change within the intervention group nor differences compared to a comparison group (20). To avoid over-interpretation of sparse data, BCTs were classified as promising if they were used in at least twice as many promising interventions as non-promising interventions (i.e., promise ratio ≥ 2), and in at least two interventions in total.

## 3 Results

### 3.1 Screening process

In total, 13,617 publications were retrieved via database search [LM]. Of those 4,045 duplicates and 1,062 publications with ineligible document types, like reviews or anthologies were removed [LM]. The remaining 8,510 titles were screened (LM, MLG, MR, SLL, TMC), of which 7,948 were excluded. This resulted in an abstract screening of 562 studies (LM, MLG, MR, SLL, TMC), of which 464 studies were excluded. We then screened 98 studies by full text (LM, MLG, MR, SLL, TMC). Of these, 68 studies were excluded. An additional five studies were excluded after the screening process. Four of them reported on a category (e. g. flood adaptation or clothing sufficiency) no other study reported on, so that comparability was not possible. One was a follow-up study that we merged with the origin study. In the end, 25 studies were included in the review. The screening process is depicted in Figure 1.

### 3.2 Study characteristics

The 25 studies included data from 14 countries on six continents. Fourteen studies were conducted in Europe (34–47), five in Asia (48–52), three in Australia (53–55) and one each in North America (56), South America (57) and Africa (58).

The different outcomes of the included studies can be assigned to the categories energy consumption [*n* = 17, (34, 36–40, 42, 46–53, 56, 58)], mobility [*n* = 8, (35, 36, 38, 41, 43, 54, 56, 57)], and water consumption [*n* = 3, (44, 45, 55)]. Three of the studies included two categories and are therefore assigned twice (36, 38, 56).

Sample sizes varied between 16 and 4,358 participants. Most studies had less than 100 [*n* = 8, (35, 39, 43, 47, 48, 53, 55, 56)] or between 100 and 499 participants [*n* = 12, (34, 36, 38, 42, 45, 46, 49–52, 54, 58)]. Three studies had 500 to 999 participants (41, 44, 57), two studies had more than 1,000 (37, 40). Recruitment happened either via advertising (*n* = 11) or via invitation (*n* = 15). One study used both methods (43).

Male and female participants were included in 14 studies (34, 37, 38, 41–46, 52–54, 56, 57). Eleven studies did not report on sex or gender.

Mean age of participants was reported by nine out of 25 studies and ranged from 35.4 years (43, 57) to 51.07 years (54). In five studies it was between 40 and 50 years (34, 38, 44, 46, 54), in three studies between 35 and 40 years (45, 56, 57) and one study had intervention groups of both age categories (43). Five studies included only adults (41, 46, 53, 56, 57). Two studies explicitly stated to include adults as well as people under 18 years as part of the household that took part in the intervention (38, 39). Nine studies did not report on age at all (35, 36, 40, 47–49, 51, 55, 58).

Regarding socioeconomic status (SES) of the participants ten studies gave no information, and 15 studies reported on income and/or education level (34, 36–38, 41, 43, 44, 46, 50–53, 56–58).

Study characteristics are presented in Table 3.

**Table 3:** Details of included studies.

Authors	Year	Country	Study design^c^	Sample size	Primary outcome	BCT-category	QA^b^
**Energy consumption**							
Abrahamse et al. (34)	2007	Netherlands	Case–control design	189	Energy consumption (MJ); Occurence of energy-saving behaviors (frequency, quantity, occurence—yes/no)	1.Goals and planning 2. Feedback and monitoring 5. Natural consequences 6. Comparison of behavior	Weak
Bardsley et al.^a^ (36)	2019	UK	Controlled field experiment with matched treatment and control areas	153	Energy consumption	4. Shaping knowledge 5. Natural consequences 6. Comparison of behavior 12. Antecedents	Strong
Bonan et al. (37)	2021	Italy	Field experiment	4,385	Energy consumption (kWh)	2. Feedback and monitoring 6. Comparison of behavior	Moderate
Büchs et al.^a^ (38)	2018	UK	Longitudinal field experiment	218	Energy consumption (kWh)	3. Social support 5. Natural consequences 6. Comparison of behavior	Weak
Erell et al. (48)	2018	Israel	Interventional, prospective case–control design	90	Energy consumption (kWh)	2. Feedback and monitoring 4. Shaping knowledge 6. Comparison of behavior	Moderate
Fijnheer et al. (39)	2021	Netherlands	Pre-post design, case–control	18	Energy consumption (kwH and m^3^ gas)	1. Feedback and monitoring 4. Shaping knowledge 6. Comparison of the behavior 8. Behavioral practice/rehearsal	Moderate
Ghesla et al. (40)	2019	Germany	Field experiment	1,345	Energy consumption (kWh)	1. Goals and planning 2. Feedback and monitoring 4. Shaping knowledge 5. Natural consequences 10. Reward and threat	Weak
Grabow et al.^a^ (56)	2018	USA	Pre-post design	16	Energy consumption (kWh, therms, CO_2_); CO_2_ emission quantity	2. Feedback and monitoring 5. Natural consequences 8. Repetition and substitution 9. Comparison of outcomes	Moderate
Hall et al. (53)	2013	Australia	Pre-post design	79	Different energy saving actions quantity	1. Goals and planning 3. Social support 4. Shaping knowledge 6. Comparison of behavior 9. Comparison of outcomes 10. Reward and threat	Weak
He & Kua (49)	2013	Singapore	Pre-post design, case–control	151	Energy consumption (kWh)	4. Shaping knowledge 5. Natural consequences 6. Comparison of behavior 7. Associations	Weak
Lu et al. (50)	2018	China	Experiment	116	Energy consumption (kWh)	4. Shaping knowledge 7. Associations	weak
McCalley et al. (42)	2011	Netherlands	Experiment with a 2 × 2 between-subjects full-factorial design	121	Energy consumption (kWh)	1. Goals and planning 2. Feedback and monitoring	weak
Mi et al. (51)	2020	China	Controlled field experiment	134	Energy consumption (kWh)	2. Feedback and monitoring 5. Natural consequences 6. Comparison of behavior	Moderate
Shen et al. (52)	2020	China	Field experiment	135	Energy consumption (kWh)	2. Feedback and monitoring 5. Natural consequences 6. Comparison of behavior 7. Associations 8. Repetition and substitution 9. Comparison of outcome 10. Reward and threat	Moderate
Thondhlana and Kua (58)	2016	South Africa	Field quasi-experiment	103	Energy consumption (kWh)	2. Feedback and monitoring 4. Shaping knowledge 5. Natural consequences 7. Associations 8. Repetition and substitution	Weak
van der Werff et al. (46)	2019	Netherlands	Experimental design	103	Switching off of appliances when not used (frequency)	1. Goals and planning 5. Natural consequences	Weak
Wemyss et al. (47)	2018	Switzerland	Experimental design	91	Energy consumption (kWh)	1. Goals and planning 2. Feedback and monitoring 3. Social support 4. Shaping knowledge 6. Comparison of behavior 10. Reward and threat	weak
**Water consumption**							
Tiefenbeck et al. (44)	2018	Switzerland	Framed field experiment	620	Mean water use per shower (l); Mean baseline shower time (s); Mean water flow (l/min); Energy consumption (kWh) while showering	2. Feedback and monitoring 5. Natural consequences 6. Comparison of behavior 12. Antecedents	Strong
Tijs et al. (45)	2017	Netherlands	Field experiment, 2 (time: pre vs. post) × 2 (appeal: monetary vs. environmental) mixed design with repeated measures on the first factor	224	Showering frequency	1. Goals and planning 5. Natural consequences 7. Associations 13. Identity	weak
Willis et al. (55)	2010	Australia	Retrofit study	44	Shower duration, volume, flow rates	2. Feedback and monitoring 12. Antecedents	Weak
**Mobility**							
Ahmed et al. (35)	2020	Belgium	N/A	52	Mode of mobility quantity (quantity of: car use; public transport use; distance travelled using active travel modes in comparison to other modes)	1. Goals and planning 2. Feedback and monitoring 5. Natural consequences 6. Comparison of behavior	Weak
Bardsley et al.^a^ (36)	2019	UK	Controlled field experiment with matched treatment and control areas	153	Car use frequency; Flight quantity and duration	4. Shaping knowledge 5. Natural consequences 6. Comparison of behavior 12. Antecedents	Strong
Büchs et al.^a^ (38)	2018	UK	Longitudinal field experiment	218	Car use quantity; CO_2_ emission reduction activity frequency	3. Social support 5. Natural consequences 6. Comparison of behavior	Weak
Diniz et al. (57)	2015	Brazil	Pre-post design, case–control	876	Bike use (yes/no)	5. Natural consequences 6. Comparison of behavior 8. Repetition and substitution	Moderate
Grabow et al.^a^ (56)	2018	USA	Pre-post design	16	Mode of mobility frequency/quantity	2. Feedback and monitoring 5. Natural consequences 8. Repetition and substitution 9. Comparison of outcomes	Moderate
Kruijf et al. (41)	2018	Netherlands	Longitudinal design	547	Bike use (quantity)	10. Reward and threat	Moderate
Ma et al. (54)	2017	Australia	N/A	313	Mode of mobility: car, bus, walk (frequency, time, distance)	1. Goals and planning 4. Shaping knowledge 8. Repetition and substitution 15. Self-belief	Moderate
Moser et al. (43)	2019	Switzerland	Field quasi-experiment	82	Mode of mobility frequency	2. Feedback and monitoring 9. Comparison of outcomes 10. Reward and threat	Weak

a Study included energy consumption as well as mobility as categories and therefore will be listed in both categories.

b Quality assessment.

c According to the authors.

### 3.3 Frequency of used behavior change techniques

We identified 29 out of 93 BCTs across all interventions. On average, an intervention employed 4.4 BCTs (median = 4; min. = 2; max. = 9). A detailed list about the frequency of application of individual BCTs in each category and the applied BCTs per intervention is provided in Supplementary Tables S1, S2.

**Figure 2:**
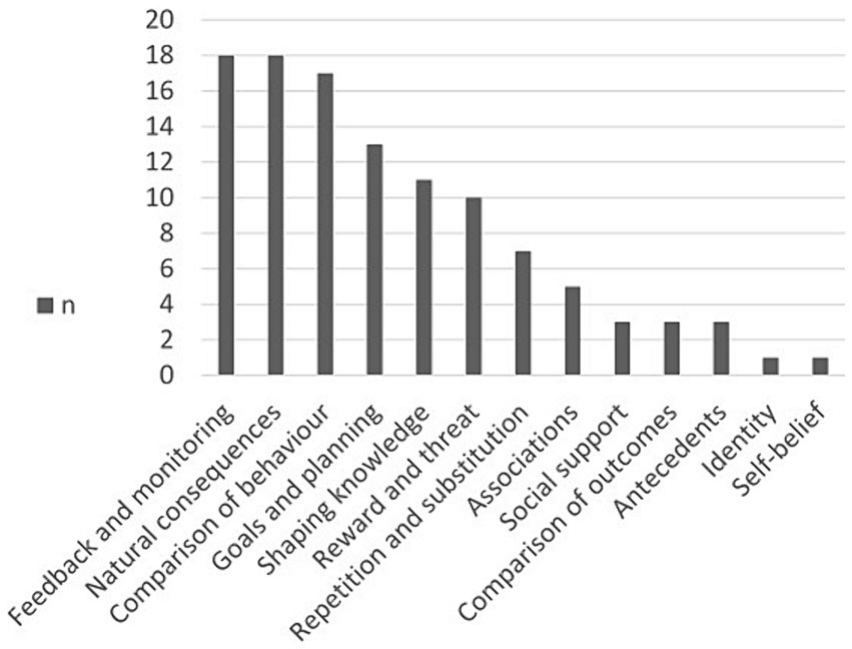
Frequenc y (*n*) of behavior change technique categories.

These BCTs cover 13 out of 16 BCT categories. Of the total amount of assigned codes (*n* = 110), 16% (*n* = 18) belong to the BCT category *natural consequences*, 16% (*n* = 18) to *feedback and monitoring*, 15% (*n* = 17) to *comparison of behavior*, 12% (*n* = 13) to *goals and planning*, 10% (*n* = 11) to *shaping knowledge*, 9% (*n* = 10) to *reward and threat*, 6% (*n* = 7) to *repetition and substitution*, 5% (*n* = 5) to *associations*, 3% (*n* = 3) to *social support*, 3% (*n* = 3) to *comparison of outcomes*, 3% (*n* = 3) to *antecedents*, 1% (*n* = 1) to *identity*, and 1% (*n* = 1) to *self-belief* (see Figure 2).

The most used individual BCTs were *Feedback on outcome(s) of behavior* (*n* = 14), *Information about social and environmental consequences* (*n* = 12), *Instruction on how to perform a behavior* (*n* = 11) and *Social comparison* (*n* = 11). Other codes were used six times or less.

### 3.4 Promise ratio

In very promising interventions the number of BCTs ranged from 1 to 6, in quite promising interventions from 2 to 8 and in the five non-promising interventions from 2 to 4 (see Table 4). There was no significant relationship between the number of BCTs used and the promise rating of interventions (F [2,24] = 1.09, *p* = 0.35). Although (very and quite) promising interventions used more BCTs (very promising: *m* = 3.90, SD = 1.37; quite promising: *m* = 4.00, SD = 1.94) than did non-promising interventions (*m* = 2.80, SD = 0.84), the difference was not significant (*t* [22] = 1.47, *p* = 0.155).

**Table 4:** Frequency of BCT category stratified by very, quite, and non-promising interventions and promise ratios.

Behavior change technique category (*n* = 25 Studies)	Promising			Non-promising	Promise ratio
	Very (*n* = 10)	Quite (*n* = 10)	Sum (*n* = 20)	(*n* = 5)	
1. Goals and planning	3	5	8	1	8
2. Feedback and monitoring	6	6	12	3	4
3. Social support	1	1	2	1	2
4. Shaping knowledge	3	6	9	1	9
5. Natural consequences	5	5	10	3	3
6. Comparison behavior	7	5	12	3	4
7. Associations	2	3	5	0	NA
8. Repetition and substitution	4	2	6	1	6
9. Comparison of outcomes	0	2	2	1	2
10. Reward and threat	3	3	6	0	NA
12. Antecedents	1	1	2	1	2
13. Identity	1	0	1	0	NA
15. Self-belief	0	1	1	0	NA

NA = not applicable.

The techniques *associations, reward and threat, identity* and *self-belief* were unique in the very and quite promising interventions. The promise ratio (PR), which gives an indication of the effectiveness of the contribution of a specific BCT to an intervention was highest for the BCTs *shaping knowledge* (PR = 9) and *goals and planning* (PR = 8) followed by *repetition and substitution* (PR = 6) (see Table 4). For the BCTs *feedback and monitoring* and *comparison of behavior* a PR of 4 was calculated, and a PR of 3 for *natural consequences.* The BCTs *social support*, *comparison of outcome* and *antecedents* were only used for three interventions each, resulting in a PR = 2.

### 3.5 Outcomes and effectiveness of interventions differentiated by category

Each intervention study was assigned to a category (i.e., energy consumption, water consumption, and mobility). The behavior change outcomes as well as the intervention techniques, categorized with the BCTTv1, will be presented in this section according to the assigned category. The classification of whether the intervention was very, quite or non-promising based on the definition by Gardner et al. (20) is also presented. Table 3 gives additional context to the studies.

#### 3.5.1 Water consumption

This category includes three studies (44, 45, 55). The particular observed behavior is shower frequency (45), shower duration, water use or flow rate (44, 55).

Two of the studies implemented a very promising (44, 45) and one study a quite promising (55) intervention. As per our BCT-coding, the intervention strategy by Willis et al. (55) is based on *feedback and monitoring* as well as *antecedents* (i.e., digital shower meter) to reduce shower duration, water volume and flow rate in the study group. The pre-post-comparison showed a significant reduction in mean shower time (mean difference: 1.34 min, *p* < 0.05), water volume used (mean difference: 15.40 L, *p* < 0.05) as well as flow rate (mean difference 1.00 L/min, *p* < 0.05). The authors state that most of the participants who already had short shower durations (i.e., below 5 min) further reduced their time while long shower durations could not be reduced (55). The second study compared duration and shower frequency in a pre-post-design using the intervention techniques *goals and planning, natural consequences* (economic vs. financial consequences), *associations,* and *identity* but without significant effects. Only a subgroup analysis revealed that one condition (environmental consequences) had a significant impact on reducing shower frequency in a four-day period [Mean: 2.98 times (pre); Mean: 2.67 times (post); *p* < 0.01] (45). The third study compared the intervention with a control group. We found that *feedback and monitoring, natural consequences, comparison of behavior* and *antecedents* regarding shower behavior (i.e., shower time, flow rate average temperature) were applied. As a result, in all domains studied significant reductions were found (shower time: −51 s, *p* < 0.01; flow rate: −0.2 L/min, *p* < 0.05; average temperature: −0.32°C, *p* < 0.05) (44).

#### 3.5.2 Mobility

The category mobility was addressed in eight intervention studies (35, 36, 38, 41, 43, 54, 56, 57) with the aim to change the mode or frequency of mobility and to reduce greenhouse gas emissions. The interventions of the category mobility are predominated by the BCT categories *natural consequences, comparison of behavior* and *reward and threat.*

There were four very promising interventions (36, 41, 43, 57) which promoted a significant shift to alternative mobility compared to the baseline and a comparator. Two of the studies promoted bicycle use for commuting (41, 57). In the first intervention study with the techniques *reward and threat* the shift from car use to e-cycling was significantly fostered (baseline of 100% car users to 68% e-cycling six months after start) (41). The second intervention study (57) on bicycle-use was based on *natural consequences, comparison of behavior* and *repetition and substitution*, as per our coding, and showed no significant difference between intervention and control group after the intervention (bike use: Intervention: *n* = 208 (47.5%), Control: *n* = 184 (42.0%), *p* = 0.10). The other three studies aimed to increase the use of various alternative means of transport for everyday routes. One analyzed if *reward and threat*, *feedback and monitoring* and *comparison of behaviors* may lead to more frequent bike use in sports club teams (43). The effect of a more frequent bicycle use in the teams was significant during the intervention in comparison to baseline (*F*(2) = 3.62, *p* < 0.05). However, the effect was only temporary and car use increased again after the intervention (43). In the fourth very promising study we identified the techniques *antecedents*, *shaping knowledge*, *natural consequences* and *comparison of behavior* (36). The intervention did not show any difference between the intervention and control group, yet revealed in both groups significant pre-post differences (vehicle use in intervention group at baseline: Mean: 21,966 kWh (SE: 3,171) and in year 3: Mean: 14,907 kWh (SE: 2,014); *p* = 0.02) (36).

Two quite promising interventions (35, 54) revealed a significant pre-post effect on mobility. The first study (54) examined changes in transportation choice behavior, i.e., reduced car use, and increased bus and walking trips between an intervention and control group. The intervention techniques we identified were *goals and planning, self-belief, shaping knowledge* and *repetition and substitution*. As a main result the intervention significantly increased walking trip time (Mean increase: 3.18 min, SD: 7.70 min, *p* < 0.05) as well as walking distance (Mean increase: 0.39 km, SD: 0.71 km, *p* < 0.01) (54). The second study focused on influencing the mode of transportation quantity using *goals and planning, feedback and monitoring, natural consequences* and *comparison of behavior* as techniques (35). The pre-post comparison in the intervention group showed significant differences for aspects of individual travel behavior, i.e., decreased car dependency (Cohen’s d = 0.28) and increased active mobility such as walking and cycling (Cohen’s d = 0.45).

Two non-promising interventions also intended to reduce car usage and increase active mobility but could not reveal significant effects (38, 56). The intervention techniques we identified were *social support*, *natural consequences* and *comparison of behavior* (38) and *feedback and monitoring*, *natural consequences*, *repetition and substitution* and *comparison of outcomes* (56).

#### 3.5.3 Energy consumption

More than half of the included studies (*n* = 17) assessed interventions regarding behavior change in energy consumption, i.e., gas and electricity use (34, 36–40, 42, 46–53, 56, 58). The predominantly used intervention techniques in this category were *feedback and monitoring* (*n* = 11), *comparison of behavior* (*n* = 11), *shaping knowledge* (*n* = 10) and *natural consequences* (*n* = 10).

Five of the interventions were rated as very promising (34, 36, 39, 47, 58). The first study applied the BCT categories *feedback and monitoring, shaping knowledge, comparison of behavior* as well as *repetition and substitution*, which resulted in total energy reduction in the intervention group (gas and electricity was measured, no proof of significance given). The difference between intervention and control group was also significant (Mean difference of energy savings: 7.9%, t (16) = −1.83, *p* < 0.05) (39). The second intervention applied *goals and planning*, *reward and threat*, *feedback and monitoring*, *comparison of behavior*, *shaping knowledge* and *social support* (47). This led to a significant energy reduction effect compared to the control condition (*F*(2, 85) = 5.02, *p* = 0.009) (*ibid.*). The third study used the BCT categories *goals and planning, feedback and monitoring, natural consequences* and *comparison of behavior.* Households exposed to the interventions saved significantly more energy compared to the control group (*F*(2,186) = 9.02, *p* < 001) (34). The fourth study implemented the BCT categories *feedback and monitoring*, *shaping knowledge*, *natural consequences*, *associations* and *repetition and substitution* (58). This led to significant energy reduction in the intervention group (Mean reduction: −24.50 kWh, *p* < 0.05). The reduction was higher than in the control group, however no effect size was reported in the study (58). The fifth very promising intervention in this category used the intervention techniques *antecedents, shaping knowledge, natural consequences* and *comparison of behavio*r (36). The study revealed differences in the pre-post comparison between the intervention and control group, respectively (electricity use in intervention group at baseline: Mean: 15.0 kWh (SE: 1.29) and in year 3: Mean: 12.0 kWh (SE: 0.95); *p* < 0.01), yet there were no significant differences between intervention and control group (36).

Seven interventions were classified as quite promising and promoted significant energy savings either compared to baseline or a comparator (40, 42, 49–53). In the first study (52) we found seven techniques that were applied in their intervention (i.e., *feedback and monitoring*, *natural consequences*, *comparison of behavior*, *associations*, *repetition and substitution*, *comparison of outcome*, *reward and threat*). They stated that a significant reduction of energy consumption was achieved within the different intervention groups as well as between the control and intervention group (no effect estimates in study) (*ibid.*). The second (42) and third study (50) each applied two strategies: *goals and planning* and *monitoring and feedback* and *shaping knowledge* and *associations*, respectively. The second study showed a significant effect between the two conditions (*F*(1, 116) = 15.93, *p* < 0.001, ⴄ^2^ = 0.12) (42). The third study revealed significant pre-post reductions (mean energy consumption reduction: 225.63 kWh, *p* < 0.001) (50). The fourth quite promising intervention study (51) used *feedback and monitoring, natural consequences* and *comparison of behavior* and showed a significant effect in the pre-post comparison of the information and environmental contribution feedback (mean percentage of electricity saved: 29–49%, *p* < 0.05). In the fifth study (40) we coded the intervention strategies *goals and planning, feedback and monitoring, shaping knowledge, natural consequences* and *reward and threat*. Their analyses revealed a significant influence of the intervention on energy consumption (Constant: 10.48, Beta (intervention arm 1): −3.5, *p* < 0.05; Beta (intervention arm 2): −0.47, *p* < 0.01; R-squared = 0.93). The sixth quite promising intervention (53) used *goals and planning*, *social support*, *shaping knowledge*, *comparison of behavior*, *comparison of outcomes* and *reward and threat* and showed an increase in energy saving behaviors in the intervention group (Mean number of activities at baseline: 9.49 (SD = 3.90), Mean number of activities post intervention: 14.04 (SD = 0.52), *p* < 0.001). The seventh intervention applied the techniques *shaping knowledge*, *natural consequences*, *comparison of behavior* and *associations* (49). Electricity consumption decreased significantly in the intervention group from baseline to post-intervention (Mean decrease: 2.21 kWh *per capita* per day, *p* < 0.01) (49).

Five interventions were rated as non-promising, because they did not foster any significant energy savings, neither in the intervention groups in a pre-post comparison nor compared to a control group (37, 38, 46, 48, 56). One study (48) used the three most frequently used techniques in the energy category: *feedback and monitoring*, *shaping knowledge* and *comparison of behavior.* The second (37) and third study (46) used only two techniques each, *feedback and monitoring* and *comparison of behavior* and *goals and planning* and *natural consequences,* respectively. The fourth study (38), using *social support*, *natural consequences* and *comparison of behavior,* did not reveal any significant changes either and was therefore one of the non-promising interventions. The fifth study (56) used the techniques *feedback and monitoring*, *natural consequences*, *repetition and substitution* and *comparison of outcomes.* It could not reveal any significant behavior change effect of the intervention.

### 3.6 Methodological quality

All 25 studies were assessed regarding the six EPHPP quality domains, as described in the methods section (see chapter 2.3). A total of 14 studies (56%) received a “weak” overall quality rating (34, 35, 38, 40, 42, 43, 45–47, 49, 50, 53, 55, 58). Nine studies (36%) received a “moderate” overall quality rating (37, 39, 41, 48, 51, 52, 54, 56, 57) and only two studies (8%) a “strong” overall quality rating (36, 44). Selection bias was mostly rated ‘moderate’ if not ‘weak’ because of widespread unclarity concerning the response rates, although almost all samples were judged to be reasonably to fully representative of the respective target population. Most authors provided adequate information on study design and randomization, but the randomization procedure was rarely described. Three of the studies had a randomized controlled trial or controlled clinical trial design, while all others had either cohort analytic (two group pre-post) or interrupted time series designs. Lack of information about potential confounders between groups and whether and how they were controlled, and also the validity and reliability of data collection methods, lead to ‘weak’ ratings for about half of the studies in these categories. Only “weak” and “moderate” ratings were given for blinding, as hardly any information was provided on whether the outcome assessors or the study participants themselves were aware of participants’ intervention status. In slightly more than a quarter of the studies, the authors did not report on withdrawal and drop-out rates, resulting in a ‘weak’ rating. When withdrawal and drop-out rates were reported, however, they were mostly strong. The results of the quality assessment are presented in the Supplementary Table S3.

## 4 Discussion

The aim of this review was to investigate which intervention techniques, represented through Behavior Change Techniques (BCTs), have been used and proved promising in interventions to promote climate-friendly behavior of individuals and households. To our knowledge, this is the first review on climate mitigation and adaptation behavior change using the Behavior Change Technique Taxonomy (BCTTv1).

Our results show that several intervention strategies to promote climate-friendly behavioral changes in the categories energy, water and mobility were effective. The BCT categories *feedback and monitoring*, *shaping knowledge*, *natural consequences*, and *comparison of behavior* are part of more than a third of the 25 included studies, however, the most effective BCTs according to their frequency in promising studies, as indicated by the promise ratio, seem to be *goals and planning*, *shaping knowledge* and *repetition and substitution*.

The setting or agreement on a goal for a behavior or an outcome, commitment, as well as the prediction of barriers or facilitators are part of *goals and planning* (31). Consistent with our results, reviews on energy consumption behavior as well as a review study by Nisa et al. (10), who analyzed RCTs of climate-friendly behavior interventions for households, found *goal setting* (18, 55) and *commitment* (17, 55) to be effective. However, they state that the effect of commitment is to be considered with caution as giving up commitment leads to exclusion of the study in RCT studies. Homburg et al. (59) found that if they randomly selected a person who received *instruction, commitment* or *goal setting* as an intervention technique, they are 54–75% more likely to exhibit climate-friendly behavior during the study period than a randomly selected person who did not receive this intervention. They labelled one effective technique *instruction*. The BCT equivalent is *instructions on how to perform the behavior* in the category *shaping knowledge* (31) that we found to be particularly effective. Wynes et al. (60) also found *instructions* to be effective. They used the categorization by Osbaldiston and Schott (61) who combined different wordings of reviews (e.g., information, knowledge, persuasion) under the label *instructions.* Nevertheless, they found a rather small effect of instructions in their meta-analysis (61). Rau et al. (12) recommend a combination of techniques involving education, training and feedback. The word *training* seems to be similar to *instructions on how to perform the behavior*. *Repetition and substitution* was mainly labelled for one of its defining techniques in this review, i.e., *behavioral practice/rehearsal* (31). Particularly this technique has proven to be part of habit formation and therefore a sustained behavior change (62). Surprisingly, there are only few studies supporting our findings (63, 64). Possibly, the identification of such studies is more difficult due to different wording.

There is the above-mentioned evidence which supports our findings, yet there are studies that highlight other techniques to be most effective. The strongest effects in the review of Nisa et al. (13) were found for *nudges/choice architecture* and *social comparison*. The former as such are not described in the BCTTv1 but could be attributed to the BCT *restructuring the physical environment* (31). Other studies found *social influence* techniques (26, 65), *feedback* (24, 25, 60), *gamification*, *community-based techniques* (25), and *appliance labeling* (65) to be effective techniques. It becomes clear that category systems or labelling of intervention techniques in intervention studies have some overlaps (e.g., *goal setting, commitment, feedback*), but also have differences in wording, definition, or degree of detail. Nonetheless, it seems that *goals and planning*, *shaping knowledge* and *repetition and substitution* might be recommendable basic components for behavior change interventions.

### 4.1 Characteristics of included studies

In regard to the characteristics of the identified studies, there are several points worth noting. First, the continents from which the studies originate show a fairly uneven distribution. As reported, most of the identified studies were conducted in Europe, a few in Asia and Australia, and only one in North America, South America, and Africa, respectively. Although the historically low *per capita* CO_2_ emissions in South America and Africa may partly explain the low number of studies, North America has, and historically has had, comparatively high *per capita* CO_2_ emissions (66) and is still only represented by a single study. The results are hence only directly generalizable to interventions targeting individuals and households in high- and upper-middle-income countries, primarily from Europe.

The behavioral outcome categories targeted by the included intervention studies were also very unevenly distributed. Most studies targeted energy consumption, about a third targeted mobility, and a few targeted water consumption. This is in line with the findings of Wynes et al. (60) and Rau et al. (12), who also identified more interventions targeting energy consumption than interventions targeting other outcome categories combined. This phenomenon might be due to high feasibility and easy outcome monitoring. Household energy conservation measures are described as straight-forward and easy to perform (60, 67). Saving energy by lowering shower water or air temperature by a few degrees might be associated with lower cost or less effort for adaptation and maintenance than trading car rides for bike rides, for example, for both the participant and the one measuring the changes (67).

Lastly, the goal of this study was to review mitigation and adaptation behavior change interventions. However, we only found one study with an intervention targeting climate change adaptation behavior using our search criteria, which ultimately had to be excluded as well. Given that climate change is now inevitable (1), more research focusing on resilient adaptation measures would likely prove helpful. Generally speaking, more research in regard to climate-friendly behaviors, preferably with a focus on behaviors with the most impact, such as mobility and energy behaviors (8, 68–70), will be needed.

### 4.2 Quality of included studies

The quality of the studies we identified in this review leaves room for improvement. The quality ratings are mostly the result of poor overall reporting in the studies, which is common in intervention research (16, 71–76) and often prevents accurate rating. Underreported aspects include among others response rates, randomization procedures, potential confounders and whether and how they were controlled, validity and reliability of data collection methods, blinding, and withdrawal as well as dropout rates. In addition, a lot of the studies do not meet basic reporting standards in regard to the study population which affects comparability.

The vast majority of the intervention studies employed cohort analytic (two group pre-post) or interrupted time series designs. The remaining three studies used randomized controlled trial or controlled clinical trial designs, which have been found to be lacking in climate-friendly intervention research (60). These are generally appropriate study designs to test mitigation and adaptation interventions, however there are some design aspects that could be improved. For one, more randomization in regard to the group allocation would be preferable for more robust study results. Furthermore, about one third of the studies used control groups that received different interventions or did not use a control group at all. To truly determine intervention components that reliably change climate-friendly behavior more studies with control groups that did not receive the intervention are essential. Lastly, the implementation of longitudinal designs with follow-up measures is needed for interventions targeting climate-friendly behavior, as potentially promising effects do not necessarily persist beyond the intervention period (13). However, a lack of follow-up measures in intervention studies targeting climate-friendly behavior has been noted (60). Of the studies reviewed here, seven used longitudinal designs (longer than 12 months), and only five studies collected follow-up data after the end of the intervention period. Consistent with the findings of Nisa et al. (13), positive intervention effects were partially sustained at follow-up in only one of these five studies (54), whereas the others reported non-significant or non-sustained effects (38, 43, 47, 56).

### 4.3 Strengths and limitations of this review

The strengths of this systematic review lie in a number of different aspects. For one, its focus on areas of daily life where climate-friendly changes are particularly difficult to realize is to be emphasized, as these are relevant levers and targets for research and practice. In addition, this review mainly includes households as intervention participants, which gives the interventions a realistic setting, as most significant changes in climate-friendly behavior affect the entire household or require its participation and hence underlines its relevance for practice. To ensure that our review covers a substantial amount of existing research, we used the five largest and most relevant databases in our literature search, spanning different key disciplines. The intervention components were described using a proven standardized instrument, the Behavior Change Taxonomy, and additionally analyzed by calculating promise ratios for each BCT to determine their success.

Even though the BCTTv1 was developed primarily for intervention design, reporting, and replication, the authors were well aware of its potential use in systematic reviews (15, 77) as “a reliable method for extracting information about intervention content, thus identifying and synthesizing discrete, replicable, potentially active ingredients (or combinations of ingredients) associated with effectiveness” (15). Besides the other advantages, using the BCTTv1 allowed us to compare studies targeting the same behavior change domains, which would otherwise be difficult to compare, by focusing on the BCTs that were used in the interventions rather than the varying outcome measures employed.

Although the BCTTv1 has its strengths, such as easier classification and greater comparability, the use of the system in this study and its consequences for the interpretation of the results need to be discussed. For one, as already mentioned, the studies found in this review and interventions in general are often described in insufficient detail (16, 71–76). This makes it more difficult to discern specific BCTs (78, 79). This means, on the one hand, that it is likely that not all intervention techniques that have actually been delivered by the researchers were identified with the BCTTv1 and, on the other hand, that it is possible that the identified BCTs might not entirely match the actual interventions. The lack of detail in intervention descriptions, however, is a problem that other reviews using other standardized or non-standardized classification systems will inevitably also encounter and do not have a standardized way to deal with, thus adding to the problem.

Another important aspect to consider when evaluating BCTs is that it is difficult to single out the effects of individual BCTs. One reason for this is, that each of studies reviewed used at least two BCTs in their intervention programs. The use of multiple interacting components in intervention research is common (16, 71, 80) and using such complex interventions, i.e., using more than one BCT to target different barriers and facilitators of the behavior, has been recommended to change target behaviors (12). Such multicomponent interventions using multiple BCTs are not necessarily the more promising interventions (81). However, when combinations of techniques are based on theory, interventions may be more effective (76, 81–83). In our review, the number of BCTs per study was not significantly correlated with the promise rating of the included studies either. Therefore, although the promise ratio of the individual BCT is reported here, it is important to keep in mind that BCTs are typically used in combination and may be effective only in that specific combination. In addition, BCTs judged to be less effective here could possibly be effective in combination with other BCTs. Like Andor et al. (65) and Nisa et al. (13), we also encourage researchers to use fewer intervention components simultaneously in future studies to better differentiate the potential of individual techniques or specific combinations of techniques. If researchers wish to use combinations of intervention components, they should preferably be theory based.

Lastly, we evaluated the BCTs used in the studies outside of the broader intervention context. Even though the evaluation of the entire intervention process is important for future replication of successful interventions (80), incoherent and incomplete intervention reporting makes evaluation difficult. Assessing the context and mode of delivery of the interventions and the theory base of the combination of BCTs was beyond the scope of the present systematic review. In the future, researchers should describe interventions, their theory base, and their context and mode of delivery in more detail and consider labelling the used intervention techniques according to the BCTTv1 (15) to standardize reporting and enhance comparability, thereby minimizing research waste and improving replicability and synthesis. Researchers need to keep in mind, however, that the feasibility and effectiveness of intervention strategies also depend on the kind of behavior, intervention design, the frequency of the behavior, behavior costs, and factors influencing the maintenance (67). This means that while BCTs help with classification, transparency, and reproducibility, they may not necessarily help with applicability across populations and behaviors. Fit-for-purpose tailoring is always needed to an extent, considering factors like the nature of the target behavior and the determinants of the behavior.

Concerning the promise ratio, one aspect to bear in mind when interpreting the results is that it cannot depict the isolated effect of individual BCTs. Other BCTs, different target behaviors, populations, settings and the diverse designs and possible embedded biases cannot be factored out with this method alone. The promise ratio only indicates the ratio of BCTs in promising versus non-promising interventions and does not take into account the effect sizes. This means that the actual behavior change achieved in interventions that are labelled promising could be negligible, but still affect the promise ratio of the respective BCTs. However, this is only a concern in regard to studies with very high sample sizes. Meta-analyses and mega-analyses (which do estimate effect sizes) could offer complementary information about which interventions are most effective. Furthermore, the publication bias in research favors an overestimation of the promise ratio of the BCTs, highlighting the importance of publishing non-significant intervention results. Lastly, following Gardner et al. (20), we assessed the promise ratio of the BCTs rather conservatively, that is, only when “they were used in two or more interventions, and at least twice as many promising as non-promising interventions” (20). As a consequence, BCTs that were rarely employed in the surveyed interventions were not assessed, but could nevertheless show potential and might warrant further investigation. Despite its drawbacks, calculating the promise ratio enabled us to easily identify intervention techniques that show promise and thus warrant further and more robust investigation, as well as, to highlight research gaps.

## 5 Conclusion

A wide range of intervention techniques have been used in climate mitigation or adaptation behavior change interventions for individuals and households in upper-middle and high-income countries, but certain techniques are more frequently used within and across the intervention categories. The three most frequently used intervention technique categories, however, are not the technique categories that are most promising in terms of behavior change. Based on the currently available evidence, our recommendation for individuals, communities, municipalities, or other entities planning to implement climate change mitigation interventions is to include components that include providing concrete instructions on how to perform the desired behavior (*shaping knowledge*), setting goals and commitments (*goals and planning*), substituting undesired behavior, and practicing desired behavior (*repetition and substitution*), as interventions with these components show the most promise.

Other reviews with similar aims use different wordings, definitions, or degrees of detail in their intervention component labelling which makes comparison of results difficult. We recommend to use a standardized classification system, like the BCT taxonomy in combination with the promise ratio, which this study has shown to be a suitable tool to classify applied intervention techniques and present an indication of successful techniques. In our experience, their combined strengths clearly outweigh their limitations. However, the limitations of the included studies, concerning intervention methods and reporting standards, still severely inhibit the potential results of reviews like this one. Going forward, intervention studies targeting climate-friendly behavior should consider designing and reporting their intervention components based on the BCTTv1 definitions, to facilitate replication and synthesis.

## Data availability statement

The original contributions presented in the study are included in the article/Supplementary material, further inquiries can be directed to the corresponding author.

## Author contributions

LM: Conceptualization, Data curation, Investigation, Methodology, Visualization, Writing – original draft, Writing – review & editing, Formal analysis. SLL: Writing – original draft, Writing – review & editing, Conceptualization, Data curation, Formal analysis, Investigation, Methodology. MR: Writing – original draft, Writing – review & editing, Conceptualization, Data curation, Formal analysis, Investigation, Methodology. CH: Writing – review & editing. ML-G: Writing – original draft, Writing – review & editing, Conceptualization, Data curation, Formal analysis, Investigation, Methodology. TMC: Conceptualization, Data curation, Formal analysis, Funding acquisition Investigation, Methodology, Project administration, Supervision, Writing – original draft, Writing – review & editing.
