# Supplementary material for: Promising behavior change techniques for climate-friendly behavior change – a systematic review
Source: Front Public Health. 2024 Aug 12;12:1396958. doi: 10.3389/fpubh.2024.1396958 (PMC11345743; doi:10.3389/fpubh.2024.1396958)
Supplement: Supplementary file 1 [file Data_Sheet_1.pdf]

Supplementary Material

Promising Behavior Change Techniques for climate-friendly behavior change – A Systematic Review

Lisa Masciangelo, Susanne Lopez Lumbi, Michel Rinderhagen, Prof. Dr. Claudia Hornberg, Dr. Michaela Liebig-Gonglach, Dr. Timothy Mc Call

*** Correspondence:** Dr. Timothy Mc Call

1 Supplementary Tables

Table S1: Frequency of Behavior Change Techniques per outcome category

**Behavior Change Technique**	**Sum**
**Energy Consumption**	
1. Goals and planning	**9**
1.1 Goal Setting (behaviour)	2
1.3 Goal setting (outcome)	4
1.8 Behavioural contract	1
1.9 Commitment	2
2. Feedback and monitoring	**12**
2.2 Feedback on behaviour	1
2.3 Self-monitoring of behavior	1
2.7 Feedback on outcome(s) of behaviour	10
3. Social support	**2**
3.1 Social support (unspecified)	1
3.3 Social support (emotional)	2
4. Shaping knowledge	**10**
4.1 Instruction on how to perform the behaviour	10
5. Natural consequences	**12**
5.1 Information about health consequences	1
5.2 Salience of consequences	1
5.3 Information about social and environmental consequences	9
5.4 Monitoring of emotional consequences	1
6. Comparison of behaviour	**11**
6.1 Demonstration of the behaviour	4
6.2 Social comparison	8
7. Associations	**4**
7.1 Prompts/cues	4

Table S1: Frequency of Behavior Change Techniques per outcome category (continued)

8. Repetition and substitution	**5**
8.1 Behavioural practice/rehearsal	3
8.2 Behaviour substitution	2
9. Comparison of outcomes	**3**
9.1 Credible source	2
9.9 Comparative imagining of future outcomes	1
10. Reward and threat	**6**
10.1 Material incentive (behaviour)	1
10.3 Non-specific reward	1
10.8 Incentive (outcome)	2
10.10 Reward (outcome)	2
12. Antecedents	**1**
12.5 Adding objects to the environment	1
**Mobility**	
1. Goals and planning	**3**
1.1 Goal setting (behaviour)	1
1.9 Commitment	2
2. Feedback and monitoring	**3**
2.3 Self-monitoring of behavior	1
2.7 Feedback on outcome(s) of behavior	2
4. Shaping knowledge	**2**
4.1 Instruction on how to perform the behavior	2
5. Natural consequences	**8**
5.1 Information about health consequences	3
5.3 Information about social and environmental consequences	4
5.4 Monitoring of emotional consequences	1
6. Comparison of behaviour	**5**
6.1 Demonstration of the behavior	3
6.2 Social comparison	2
8. Repetition and substitution	**4**
8.1 Behavioral practice/rehearsal	3
8.2 Behavior substitution	1
9. Comparison of outcomes	**1**
9.1 Credible source	1
10. Reward and threat	**4**
10.1 Material incentive (behavior)	2
10.2 Material reward (behavior)	2
12. Antecedents	**1**
12.5 Adding objects to the environment	1
15. Self-belief	**1**
15.3 Focus on past success	1

Table S1: Frequency of Behavior Change Techniques per outcome category (continued)

**Water Consumption**	
1. Goals and planning	**1**
1.3 Goal setting (outcome)	1
2. Feedback and monitoring	**4**
2.2 Feedback on behavior	2
2.7 Feedback on outcome(s) of behavior	2
5. Natural consequences	**2**
5.2 Salience of consequences	1
5.3 Information about social and environmental consequences	1
6. Comparison of behaviour	**1**
6.2 Social comparison	1
7. Associations	**1**
7.1 Prompts/cues	1
12. Antecedents	**2**
12.5 Adding objects to the environment	2
13. Identity	**1**
13.5 Identity associated with changed behavior	1

Note: Some studies included energy conservation as well as mobility as categories. The BCT of these studies are therefore listed in both categories.

Table S2: Behavior Change Techniques per intervention

BCT	Study																								
	9	10	11	12	13	14	15	16	17	18	19	20	21	22	23	24	25	26	27	28	29	30	31	32	33	**Sum**
**1.1**		•											•							•						**3**
**1.3**	•						•		•			•		•												**5**
**1.8**													•													**1**
**1.9**		•											•							•	•					**4**
**2.2**									•		•											•				**3**
**2.3**																							•			**1**
**2.7**	•	•		•		•	•		•	•	•			•	•			•	•			•			•	**14**
**3.1**														•												**1**
**3.3**					•															•						**2**
**4.1**			•			•	•							•	•	•	•		•	•	•				•	**11**
**5.1**		•																					•	•		**3**
**5.2**							•				•															**2**
**5.3**	•	•	•		•							•	•			•		•	•				•	•	•	**12**
**5.4**																							•			**1**
**6.1**	•	•	•													•				•				•		**6**
**6.2**	•	•		•	•	•				•	•			•	•			•	•							**11**
**7.1**												•				•	•		•						•	**5**
**8.1**			•			•																	•	•		**4**
**8.2**																			•		•				•	**3**
**9.1**																				•			•			**2**
**9.9**																			•							**1**
**10.1**								•		•										•						**3**
**10.2**								•		•																**2**
**10.3**														•												**1**
**10.8**							•												•							**2**
**10.10**							•												•							**2**
**12.5**			•								•											•				**3**
**13.5**												•														**1**
**15.3**																					•					**1**
**Sum**	**5**	**7**	**5**	**2**	**3**	**4**	**6**	**2**	**3**	**4**	**5**	**4**	**4**	**6**	**3**	**4**	**2**	**3**	**9**	**7**	**4**	**3**	**6**	**4**	**5**	**110**

Table S3: Quality Assessment

	Selection Bias	Study design	Confounders	Blinding	Data Collection Method	Withdrawals and Dropouts	Overall Rating
Abrahamse et al.	-	+	-	+	-	+	Weak
Ahmed et al.	+	+	-	+	-	++	Weak
Bardsley et al.	+	+	++	+	++	++	Strong
Bonan et al.	-	++	++	+	++	++	Moderate
Büchs et al.	-	+	++	+	-	++	Weak
Diniz et al.	+	+	++	+	-	++	Moderate
Erell et al.	-	+	++	+	++	++	Moderate
Fijnheer et al.	+	+	-	+	++	++	Moderate
Ghesla et al.	-	++	-	-	-	++	Weak
Grabow et al.	+	+	++	-	++	++	Moderate
Hall et al.	+	+	-	+	-	-	Weak
He et al.	+	+	-	+	-	-	Weak
Kruijf et al.	+	+	++	+	++	-	Moderate
Lu et al.	+	+	-	+	-	++	Weak
Ma et al.	+	+	++	+	++	-	Moderate
McCalley et al.	-	++	-	+	-	-	Weak
Mi et al.	-	+	++	+	++	++	Moderate
Moser et al.	+	+	-	+	-	+	Weak
Shen et al.	+	+	-	+	++	++	Moderate
Thondhlana et al.	+	+	-	+	++	-	Weak
Tiefenbeck et al.	+	+	++	+	++	++	Strong
Tijs et al.	+	+	-	+	-	++	Weak
van der Werff et al.	+	+	-	-	-	-	Weak
Wemyss et al.	+	+	-	+	++	-	Weak
Willis et al.	+	+	-	+	++	-	Weak

Note: ++ = strong; + = moderate; - = weak
